# Myoneurectomy for Cribbing1Read at the Thirty-sixth Annual Meeting of the American Veterinary Medical Association, September, 1899.

**Published:** 1899-11

**Authors:** S. J. J. Harger

**Affiliations:** Professor of Anatomy and Zoötechnics, Veterinary Department, University of Pennsylvania


### REPORTS OF CASES.

### MYONEURECTOMY FOR CRIBBING.[1]

By S. J. J. Harger, V.M.D.,

PROFESSOR OF ANATOMY AND ZOÖTECHNICS, VETERINARY DEPARTMENT, UNIVERSITY OF PENNSYLVANIA.

Various contrivances for the prevention of cribbing have from time to time been invented. These, as well as the etiology, pathology, and symptoms of this abnormal act, shall here pass unnoticed. Based upon the action of some of the cervical muscles in this act, several operations upon a number of these muscles and their nerve-supply have, during the last year, with a curative purpose in view, been practised. These two operations are a resection of the sterno-hyoid and sternothyroid muscles and of the branch of the eleventh pair (spinal accessory) of cranial nerves (maxillary). One or both operations may be practised on the same subject; the latter is the usual procedure.

[1] Read at the Thirty-sixth Annual Meeting of the American Veterinary Medical Association, September, 1899.

I will not pass into the details of the technique of these operations, which are two of a general class, nor into the anatomical and physiological reasons for the same. These details I have explained in an article in a recent number of the JOURNAL OF COMPARATIVE MEDICINE AND VETERINARY ARCHIVES. The essential point is to destroy in the cribbing act the influence of the two above-named muscles and of the sternomaxillaries, the latter receiving their motor supply from the spinal accessory. Omitting the details of these operations, which you have perhaps practised and seen at the clinical demonstrations, I will pass to the more practical side—the results, such as I have found them in my experience—by reviewing a number of cases.

CASE I.—B. g., aged. Operated on July, 1898. Cribbed incessantly. Chronic; condition poor; hide-bound. Resected the sternohyoid and the sternothyroid muscles. Since the operation and up to the present time the animal is very much improved, and cribs once in a while. The general condition is good. The cure is not complete. Owner satisfied with the result.

CASE II.—R. g. Cribbing of moderate frequency. Made a resection of the same muscles in July, 1898. At last report owner said that the horse cribbed, but only about half as often, and not with the same effort and completeness.

CASE III.—B. g.; no history. Made resections of the muscles as well as of the spinal accessory nerves in January, 1899. A student informed me that for three months the horse did not crib, but afterward returned to his old habit.

CASE IV.—B. m., young thoroughbred. Performed both operations, as in Case III., February, 1899. Owner's statement September, 1899: "The mare was the worst cribber I ever saw. She would crib just as well with a strap on as off. Before you operated on her she was hide-bound and pot-bellied. She is now fat and has a good, glossy coat. Ten days after the operation she began to crib again. I put the strap on her and she tried to crib again, but could not. I kept the strap on for four months, when I took it off; since then I have not seen any indication of cribbing, and consider the operation a complete success."

CASE V.—S. g.; chronic and very persistent cribber; bloating and frequent colics. January, 1899, I performed the double operation. The cure is not quite complete, and the animal sometimes cribs. The general condition of the animal is good, and the result is satisfactory to the owner.

CASE VI.—B. m.; history and condition the same as in previous case. Performed the same operation; results indefinite. Shortly after the operation there was no change, and since then no report.

Case VII.—R. g. Cribbing incessant and chronic for at least four years; condition poor. Double operation July, 1899. There was no immediate change, but since has been improving daily, and now cribs very little.

Case VIII.—Gr. g. A very severe case, chronic and accompanied with bloating. Operation about four months ago. Did not crib for two weeks afterward; then was noticed to crib about half a dozen times daily, but very slightly. There was no more bloating, and the owner seemed satisfied.

Case IX.—Five-year-old mare. Cribbing not very bad and not chronic. Operation six weeks ago. Cribbing has ceased entirely, although the time since the operation has been rather short to form a positive conclusion.

For the last two cases I am indebted to a professional friend, Dr. Lloyd Zaner, of Wilkesbarre, Pa.

Immediately after the operation the animal makes a few ineffectual attempts to crib, and then may cease entirely for a variable period, when it may return to the habit, especially in chronic cases. Perhaps here, as in one of the cases cited, the use of the cribbing-strap for some time afterward may be of advantage, in order to increase the restraint and allow the animal time to be weaned of its old habits.

This is a short résumé of my experience, and everyone of you may draw conclusions. We should not be too sanguine until we have had more experience with this operation. The curative effect is not as great as could be desired or, perhaps, was anticipated.

From perhaps a premature stand-point the results might indicate that chronic cases may be very much relieved and the general condition of the animal improved; again, in some cases these good results may be only temporary. In young animals, or those in which cribbing is of recent date, there is a probability of a permanent cure. The experience of others is necessary before a definite conclusion can be formed.

---

Dr. John W. Adams, Professor of Surgery at the Veterinary Department of the University of Pennsylvania, is a matriculant of the Medical Department of the same institution.

